# Concomitant lithium increases radioiodine uptake and absorbed doses per administered activity in graves’ disease: comparison of conventional versus lithium-augmented radioiodine therapy

**DOI:** 10.3389/fmed.2024.1382024

**Published:** 2024-04-05

**Authors:** Fadi Khreish, Andrea Schaefer-Schuler, Leonie Roth, Caroline Burgard, Florian Rosar, Samer Ezziddin

**Affiliations:** ^1^Department of Nuclear Medicine, Saarland University Hospital, Homburg, Germany; ^2^Department of Nuclear Medicine, Campus-Fulda, University of Marburg, Fulda, Germany

**Keywords:** Graves’ disease, radioiodine therapy, lithium, dosimetry, administered activity, thyroidal radioiodine uptake, thyroid dose, efficacy

## Abstract

**Background:**

Lithium inhibits iodine and thyroid hormone release from thyroid cells, possibly increasing radioiodine retention and anti-hyperthyroid efficacy when given adjunctively to radioiodine therapy (RAI) of Graves’ disease (GD). However, the literature contains limited dosimetric data regarding the influence of concomitant lithium in this setting.

**Methods:**

We retrospectively compared dosimetric variables in patients undergoing RAI with/without adjunctive lithium (*n* = 52 each). We assessed two low-dose, short-duration oral lithium carbonate regimens, 450 mg/d (*n* = 22) or 900 mg/d (*n* = 30), for a mean of 4.7 ± 1.4 d starting upon RAI administration. Patients underwent diagnostic testing to measure thyroidal radioiodine uptake (RAIU) 24 h ± 2 h after ingesting up to 5 MBq radioiodine, receiving individualized RAI activities 24 h later. Using ≥3 RAIU daily measurements starting 24 h post-RAI, researchers were able to determine the effective radioiodine half-life and absorbed dose to the thyroid; we also calculated the absorbed dose per administered activity concentration within that organ. Rates of GD cure, defined as reaching euthyroidism or hypothyroidism post-RAI, were evaluated in patients with ~6 months or longer post-RAI follow-up.

**Results:**

The lithium dosage subgroups had similar dosimetric values and thus are considered together. Lithium patients and controls had similar average “diagnostic” RAIU (51.1% ± 15.7% vs. 50.6% ± 13.8%, *p* = 0.820), but the former had significantly higher RAIU post-RAI (56.3% ± 13.5% vs. 49.1% ± 13.5%, *p* = 0.002), reflecting significantly greater change in the former (+16.2% ± 30.4% vs. -1.8% ± 16.1%, *p* = 0.001). Radioiodine effective half-life was non-significantly longer in lithium patients (5.43 ± 1.50 d vs. 5.08 ± 1.16 d, *p* = 0.192). The mean RAI administered activity was 27% less in lithium patients (677 ± 294 MBq vs. 930 ± 433 MBq, *p* = 0.001), but GD cure rates were similar (83% [39/47] vs. 82% [33/40], *p* = 0.954), possibly due to the significantly higher thyroid dose in the lithium patients, especially in thyroid gland with a volume ≤ 20 mL (1.04 ± 0.44 Gy/MBq vs. 0.76 ± 0.30 Gy/MBq, *p* = 0.020). Day 3 serum lithium concentrations were low (450 mg/d: 0.26 ± 0.12 mmol/L, 900 mg/d: 0.50 ± 0.18 mmol/L); no lithium toxicity was noted.

**Conclusion:**

Lithium augmentation may increase the RAIU and thyroid absorbed dose, permitting potentially decreased RAI activities without sacrificing efficacy. Our observations should be confirmed in a prospective, randomized trial.

## Introduction

Hyperthyroidism caused by thyroid-stimulating hormone (TSH) receptor autoantibodies, also known as Graves’ disease (GD), occurs with an incidence of approximately 40/100,000 inhabitants per year (1, 2). Radioiodine therapy (RAI) is a well-established treatment for the condition (3, 4), especially in patients with high recurrence risk or long-standing hyperthyroidism (5). Moreover, RAI is the method of choice in cases of high surgical risk or intolerable side effects of antithyroid drugs and for patients with multiple comorbidities (6, 7). Ablative dose approaches are the standard of practice with RAI, resulting in therapeutic success defined as functional thyroid tissue ablation in 70–85% of cases (8–12).

Lithium has long been known to inhibit the release of iodine and thyroid hormones (13–16). To increase the ablation efficacy of RAI in patients with GD or other forms of hyperthyroidism, preclinical and clinical studies have suggested a potential benefit of adjunctive lithium medication (17–22). In particular, this strategy has been shown to potentially improve radioiodine retention as well as to increase success rates of RAI (16–19). Some studies also have found that lithium led to faster control of hyperthyroidism and reduced post-RAI peak serum concentrations of free triiodothyronine (fT3) and free thyroxine (fT4) (17, 21, 22). In contrast, another relatively large study (*n* = 134) could not demonstrate a higher treatment success rate from adding lithium to RAI (23). Thus, to date, there has been no strong evidence-based recommendation for concomitant use of lithium with RAI.

All these studies only examined cure rates with a very limited examination of dosimetric variables. Due to the scarce data about this latter subject, the present retrospective study sought to compare absorbed doses and radioiodine kinetic variables between patients who received lithium in addition to RAI and a historic control group given non-augmented standard RAI. As secondary endpoints, we also assessed rates of cure of GD, defined as attainment of a euthyroid to hypothyroid state.

## Subjects and methods

### Study design, treatment, and ethics

In this retrospective cohort study, the database of the Department of Nuclear Medicine at Saarland University Hospital, a tertiary referral center in Germany, was screened for GD patients given lithium as an adjunct to RAI. In November 2015, the addition of lithium to RAI was introduced at our institution, and as of this writing, 52 patients have been treated with this combination and included in this study. During the period from November 2015 to March 2019, only five patients given lithium with RAI for GD were excluded from this analysis; of these, three patients did not receive lithium due to contraindications for lithium, and two patients were excluded from this analysis because they had been treated with radioactive iodine in their previous history. In total, datasets of 104 patients treated with RAI for GD were analyzed, including the aforementioned 52 consecutive patients given adjunctive lithium and 52 patients of the control group. The latter were consecutively treated before November 2015 (2012–2015) and received standard RAI without lithium augmentation.

RAI was conducted following the guidelines of the German Society for Nuclear Medicine (5). A diagnostic test was performed pre-RAI, to assess radioiodine uptake (RAIU) of the thyroid. Antithyroid drugs were stopped at approximately 72 ± 12 h before this test. One measurement of thyroid RAIU was taken with a gamma counter (Veenstra Instruments, Joure, The Netherlands) 24 ± 2 h after ingestion of a maximum 5 MBq iodine-131 (I-131), but the procedure did not allow the determination of the effective half-life of radioiodine. Thyroid volume was determined sonographically (Samsung HM70A, Samsung Medison, Seoul, Republic of Korea) with a 7.5-MHz linear transducer, using the ellipsoid model (24). Using the Marinelli formula (25), an individual RAI activity was calculated for each patient to deliver an absorbed dose of 300 Gy. This activity was administered 24 h after completion of the diagnostic radioiodine test. The patients were hospitalized in median (minimum–maximum) 5 days (3–12).

Lithium augmentation began on the day of RAI capsule administration randomly with either 450 mg/d (22/52 patients, 42%) or 900 mg/d (30/52 patients, 58%) of lithium carbonate (Quilonum® retard, Teofarma Srl, Pavia, Italy). Lithium was given as a single daily dose for a mean period of 4.7 ± 1.4 days (4.46 days in patients who received 450 mg and 4.85 days in patients who received 900 mg/d of lithium). Three days later, the serum lithium level was determined, and treatment was continued or discontinued based on that level reaching a threshold of psychotherapeutic limit of 0.8–1.2 mmol/L.

After RAI, at least three measurements of thyroid RAIU (on day 1, day 2, and day 3 after RAI) were performed with the gamma counter (Veenstra Instruments, Joure, The Netherlands), starting 24 ± 2 h after RAI capsule administration. Here, a calibration factor is used to convert the measured count rate to activity (counts-per-minute/MBq, cpm/MBq). This calibration factor is based on measurements of a thyroid phantom filled with capsules of known activity (range: 50 MBq to 3,700 MBq), which are periodically performed as part of the quality control. The effective half-life was calculated by linear regression of the three patient measurements on day 1, day 2, and day 3 assuming monoexponential decay. At least the individual thyroid absorbed dose was calculated by the Marinelli formula (25), involving the estimated values of the uptake and the effective half-life of the patient.

Absorbed dose per administered activity D/A [Gy/MBq], where D = absorbed dose and A = treatment activity was calculated. First, we compared the absorbed dose per administered activity in the lithium vs. the control group including all patients. To account for variations in thyroid volume in a second evaluation, we compared the D/A for patients with normal thyroid gland volume (defined as volume ≤ 20 mL) vs. enlarged thyroid gland (volume > 20 mL), respectively. For this calculation, homogeneous activity concentration within the organ was assumed. According to German regulations, a planar scintigraphy of the neck was undertaken on day 3 after RAI using a single-headed gamma camera (MIE Diacam, MIE, Seth, Germany) equipped with high-energy collimators.

Patients provided written informed consent for all therapeutic modalities as well as for the use of their data in scientific reports. No ethics committee approval was required for the present analysis because it was an observational study using de-identified data.

### Thyroid function tests and clinical follow-up

Blood samples were taken for thyroid function tests, i.e., measurement of fT4, fT3, and TSH immediately prior to the diagnostic test (48 h before therapeutic capsule administration). Patients were followed for at least 6 months from the date of the RAI. Laboratory tests including free T4, free T3, and TSH were approximately performed every 2 months.

### Statistics

IBM SPSS version 25 (SPSS Inc., Chicago, IL, USA) was used for statistical analyses. To compare characteristics between the study groups (RAI + lithium, RAI alone), the unpaired *t*-test was used for normally distributed data, and the Mann–Whitney U-test for non-normally distributed data. For comparisons of lithium dosage subgroups, the paired *t*-test was used. A *p*-value of <0.05 was defined as statistically significant. *p*-values were not adjusted for multiple comparisons due to the exploratory nature of this study.

## Results

No significant differences were noted in the patient characteristics of the two study groups (Table 1); however, thyroid volume tended to be larger in the lithium patients than in the controls. Significant intergroup differences were seen in the administered radioiodine activities, which could be explained by application when calculating individualized activities (to deliver an absorbed dose of 300 Gy) of different values for the assumed radioiodine half-life in GD before 2015 (control group: 3 days, the institutional policy before 2015) vs. after November 2015 (RAI-lithium group: 4.5 or 5 days) (26, 27).

**Table 1:** Patient, treatment, and dosimetric characteristics.

	Lithium group	Control group	*p*-value
Patients (male/female)	52 (12/40)	52 (13/39)	0.819
Age [yr]	51 ± 16	52 ± 12	0.956
Baseline fT4 [ng/dL]	1.35 ± 0.39	1.61 ± 0.81	0.239
Thyroid volume [mL]	22.54 ± 13.46	19.33 ± 9.06	0.429
Administered RAI activity [MBq]	677 ± 294	930 ± 433	**0.001**
Cure* rate, % (*n*)	83% (39/47)	82% (33/40)	*p* = 0.954
Serum lithium concentration [mmol/L]			
All patients	0.40 ± 0.19	NA	NA
450 mg/d subgroup (*n* = 22)	0.26 ± 0.12^†^		
900 mg/d subgroup (*n* = 30)	0.50 ± 0.18^†^		

NA, not applicable; RAI, radioiodine therapy. ^*^Cure of Graves’ hyperthyroidism was defined as the attainment of a euthyroid or hypothyroid state. ^†^Mean serum lithium concentrations, 450 mg/d subgroup vs.900 mg/d subgroup, *p* < 0.001. *P* values given in bold type were statistically significant at *p* < 0.05.

**Figure 1:**
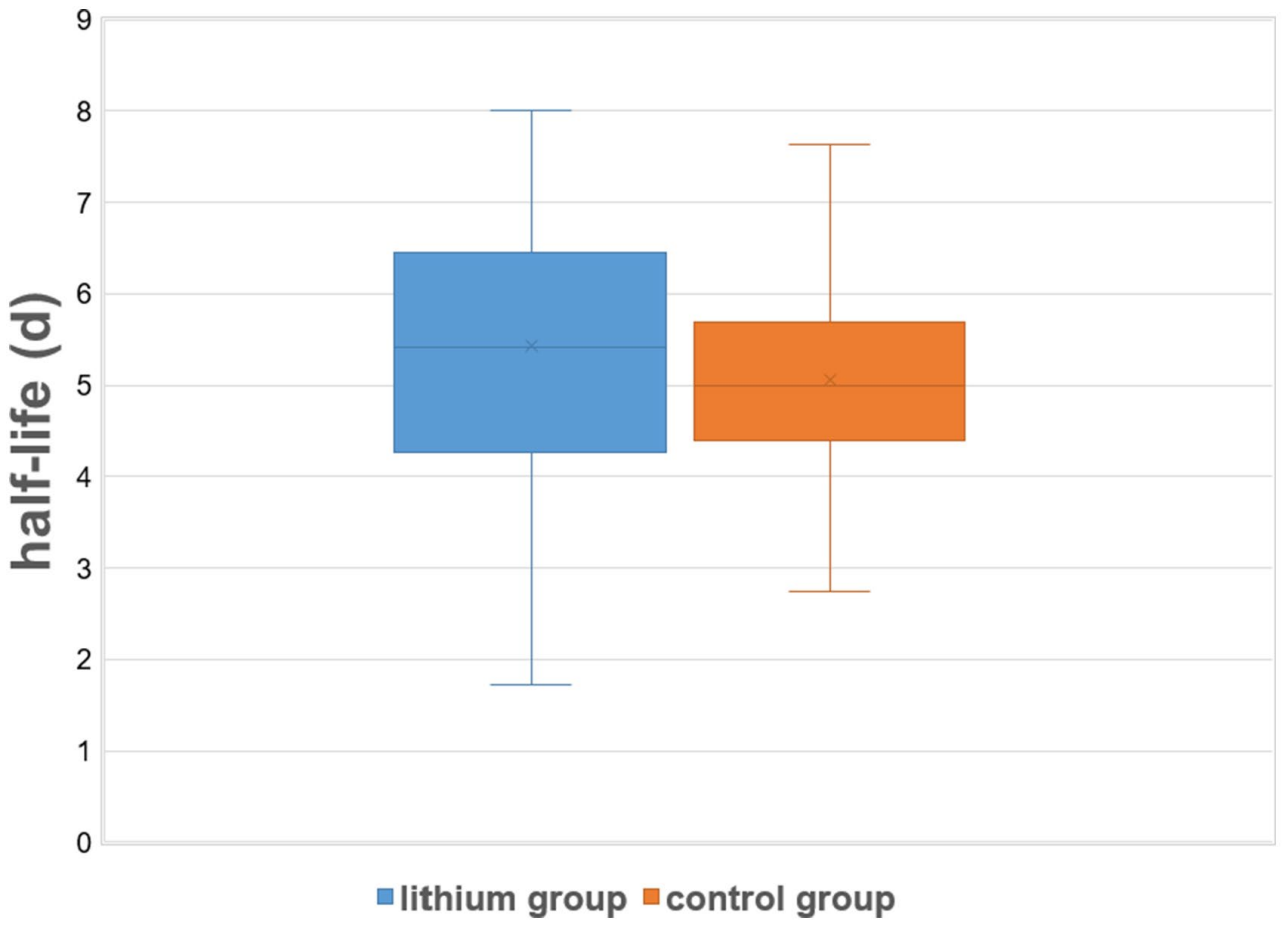
Effective intrathyroidal radioiodine half-life under therapy: control (RAI only) group vs. the lithium group. On lithium augmentation, a small increase in post-RAI effective half-life was observed (mean difference: 8.4 ± 50.4 h = 0.35 ± 2.1 d). The vertical lines show the minimum and maximum effective half-life for each study group, while the shaded areas correspond to the mean ± SD for each group.

**Figure 2:**
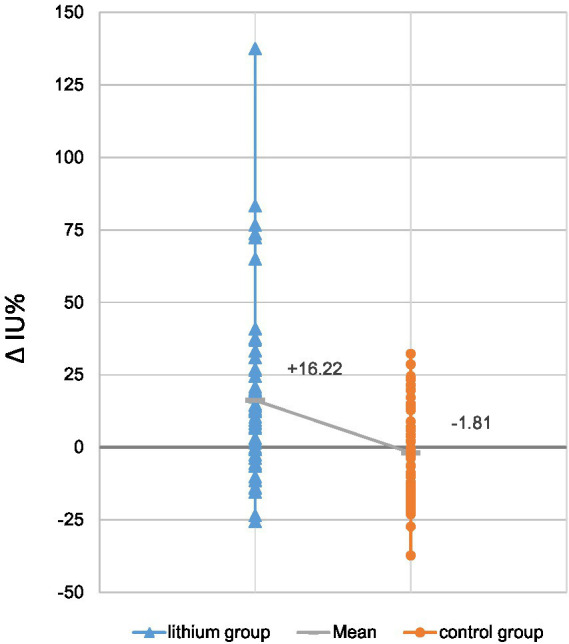
Relative changes in radioiodine uptake, post-RAI vs. diagnostic test: lithium group vs. control group (RAI only) (*N* = 52 each). The change in uptake of the control group averaged −1.81% ± 16.13%, while the lithium group had a mean increase of 16.22% ± 30.35%. Grey horizontal hatches in each bar denote the mean value for the study group; the sloping solid line between the hatches highlights the intergroup difference in these values.

Table 2 summarizes dosimetric results by treatment group. The lithium group exhibited a slightly and non-significantly longer intrathyroidal radioiodine half-life compared to the controls (Figure 1). Only seven patients (13%) from the lithium group and six patients (11%) from the control group had a half-life under 3.6 d. On average, RAIU in the diagnostic test did not differ between the two study groups. In contrast, the lithium group had significantly higher mean RAIU post-RAI (lithium group vs. the control group: 56.3% ± 13.5% vs. 49.1% ± 13.5%, *p* = 0.002). This observation reflected a significant intra-individual relative increase in uptake in the lithium group, but a slight relative decrease in the control group: 16.22% ± 30.35% vs. -1.81% ± 16.13%, *p* = 0.001 (Figure 2). After excluding the outliers in the lithium group (6 patients in the lithium group with relative change in uptake >40% were defined as outliers), the lithium group still had a significantly higher mean RAIU post-RAI (lithium group vs. control group: 56.6% ± 12.9% vs. 49.1 ± 13.5%, *p* = 0.006). This observation reflected again a significant intra-individual relative increase in uptake in the lithium group (after exclusion of the outliers), but a slight relative decrease in the control group: 7.29% ± 15.89%, vs. -1.81% ± 16.13%, *p* = 0.006.

**Table 2:** Dosimetric variables by treatment group.

Variable	Lithium group	Control group	*p*-value
Thyroid absorbed dose [Gy]	392 ± 150	464 ± 128	**0.007**
Thyroid absorbed dose per administered activity [Gy/MBq] in normal thyroid gland volume (≤ 20 mL)	1.04 ± 0.44	0.76 ± 0.30	**0.020**
I-131 half-life in thyroid tissue [d]	5.43 ± 1.50	5.08 ± 1.16	0.192
Uptake, diagnostic test [%]	51.1% ± 15.7%	50.6% ± 13.8%	0.820
Uptake post-RAI [%]	56.3 ± 13.5%	49.1% ± 13.5%	**0.002**
Absolute change of uptake [%]	5,22% ± 10,42%	−1.52% ± 8.00%	**0.001**
Relative change of uptake [%]	16.22% ± 30.35%	−1.81% ± 16.13%	**0.001**

All values for dosimetric variables are presented as mean ± SD. All dosimetric variables were calculated based on post-RAI measurements, except for the uptake of test activity. *p*-values given in bold type were statistically significant at *p* < 0.05. RAI, radioiodine therapy.

In our cohort, 30 of 52 patients in the lithium group and 30 of 52 patients in the control group had a pre-therapeutic thyroid gland volume of ≤20 mL. Our results reveal that (1) the post-RAI thyroid absorbed dose per administered activity D/A was higher in the lithium group vs. the control group, even if it was not significant (0.75 ± 0.49 Gy/MBq vs. 0.60 ± 0.32 Gy/MBq, *p* = 0,060) for the whole group. However, (2) in patients with normal thyroid gland volume (volume ≤ 20 mL), the post-RAI thyroid absorbed dose per administered activity D/A was significantly higher in the lithium group vs. the control group (1.04 ± 0.44 Gy/MBq vs. 0.76 ± 0.30 Gy/MBq *p* = 0.020). No significant differences in D/A were found in patients with thyroid volume of >20 mL (*p* = 0.560).

The mean serum lithium concentration measured on the third day after RAI was 0.26 ± 0.12 mmol/L with the 450 mg/d regimen and 0.50 ± 0.18 mmol/L with the 900 mg/d regimen. Lithium concentration appeared to have no significant influence on dosimetric variables (Table 3).

**Table 3:** Association of daily lithium dose with dosimetric variables.

	450 mg/d *n* = 22	900 mg/d *n* = 30	*p*-value
Radioiodine half-life in thyroid tissue, post-RAI [d]	5.40 ± 1.41	5.45 ± 1.59	0.905
Relative change of uptake, post-RAI vs. diagnostic test [%]	17.72% ± 33.33%	15.13% ± 28.50%	0.875
D/A [Gy/MBq], post-RAI	0.78 ± 0.54	0.72 ± 0.46	0.646

RAI, radioiodine therapy.

A cure for Graves’ hyperthyroidism achieved by RAI was defined as reaching a euthyroid to hypothyroid metabolic state following the procedure. Data from at least 6 months of clinical follow-up post-RAI were available for 47 of 52 (90%) patients in the RAI-lithium group and for 40 of 52 (77%) patients in the control group. In 39 of 47 patients (cure rate of 83%) in the RAI-lithium group, either euthyroidism (16/39, 41%) or hypothyroidism (23/39, 59%) was documented after the procedure. Similarly, either euthyroidism (15/33, 45%) or hypothyroidism (18/33, 55%) was documented in 33 of 40 patients (cure rate of 82%) in the lithium group after the procedure. This difference in cure rates between the two groups was not significant (*p* = 0.954).

## Discussion

The present study has shown for the first time that augmentation of RAI with lithium significantly increased RAIU in patients with GD. We compared pretreatment vs. posttreatment dosimetric variables in 104 patients undergoing RAI, 52 with lithium medication starting the day of radioiodine capsule administration, and 52 without lithium medication. On average, significantly (*p* = 0.001) increased uptake in the post-therapeutic measurement vs. a pre-therapeutic determination was observed in the lithium group (absolute change in percentage points: +5.22%, relative change: +16.2%), but not in the control group (absolute change in percentage points: −1.52%, relative change: −1.81%). Moreover, we found a significantly higher mean absorbed dose per administered activity in patients with normal thyroid gland volume of ≤20 mL in the lithium group than in the control group: 1.04 ± 0.44 Gy/MBq vs. 0.76 ± 0.30 Gy/MBq (*p* = 0.020). These findings suggest that augmentation of RAI with lithium may help to achieve higher uptake and therefore higher absorbed doses per administered activity, especially in normal thyroid gland volume.

No general recommendations are given for adjunctive lithium regimens in patients undergoing RAI. Some groups have proposed administering approximately 900 mg/d of the drug for periods between 6 and 21 days (16–23, 28). Bogazzi et al. (18) reported approximately 31 and 37% respective increases in absorbed dose when administering lithium, 900 mg/d, for 6 days or 19 days starting the day of RAI administration (*n* = 12 in each group). We intended to test whether similar results could be obtained upon reducing the dose of lithium therapy. Therefore, we gave either 900 mg/d (30 patients) or 450 mg/d (22 patients) for a mean of 4.7 ± 1.4 d starting the day of RAI. As expected, the mean serum lithium concentration in both subgroups remained low (0.26 ± 0.12 mmol/L with 450 mg/d, 0.50 ± 0.18 mmol/L with 900 mg/d) and hence was considered to be safe. Hence, even the higher doses resulted in mean serum concentrations of lithium well below the psychiatric limit (0.8–1.2 mmol/L) (29) and avoided side effects. The analysis of the dosage subgroups revealed similar results regarding both RAIU and radioiodine half-life (Table 2). Therefore, our lower dose lithium dosage regimen was considered feasible.

There is a well-known functional difference of RAIU in the thyroid between patients with GD and those with other benign thyroid disorders, e.g., nodular goiter, which is usually reflected by the shorter half-life of I-131 in GD (26). In our calculations of the RAI activity, the half-life of I-131 given as a “diagnostic” activity was initially assumed to be 3 days. However, our actual data collected after RAI reveal a mean thyroidal half-life of 5.08 ± 1.16 d even for the control group. This value agrees very well with values of “therapeutic half-life,” 4.7 d–5.4 d, reported in recent years (30–32). However, with lithium augmentation, only a small increase in effective half-life was observed (6.9% on average, corresponding to 8.4 h), which was statistically non-significant (*p* = 0.192). In contrast to our findings, a more marked prolongation of effective half-life by lithium has appeared in the literature. Dunkelmann et al. showed that oral lithium medication lengthened intrathyroidal RAI half-life, at least in GD patients who had a very short half-life (2.2 d) in a radioiodine test (30). These authors found increased mean half-life in post-RAI measurements vs. in the diagnostic test of approximately 10% for the “no lithium group” (3.2 d vs. 2.9 d) and over 60% for the lithium group (3.6 d vs. 2.2 d). Nevertheless, only a small number of patients (19 in the no-lithium group and 20 in the lithium group) could be included in that study, and no investigation on RAI plus lithium was performed on GD patients with longer radioiodine half-life in the thyroid in the diagnostic test.

Bogazzi et al. (19) have investigated the impact of lithium on the efficacy of RAI against GD, determining the proportion of cured patients during 1 year of follow-up in 298 patients. This group reported that patients treated with RAI plus lithium had a higher cure rate (91%) than those treated with RAI alone (85%) (*p* = 0.03). In contrast, in our study, in subgroups of patients with available follow-up data, no statistically significant difference in cure rate was found between patients treated with lithium and RAI and those treated with RAI alone (83% in the lithium group vs. 82% in the control group, *p* = 0.954). Notably, however, the groups had these comparable cure rates despite the lithium patients receiving a significantly lower mean administered radioiodine activity (677 ± 294 MBq vs. 930 ± 433 MBq, *p* = 0.001). This may possibly be due to the higher absorbed thyroid dose per administered activity, which was significant in the thyroid gland with normal volume (≤ 20 mL), on lithium augmentation (1.04 ± 0.44 Gy/MBq for lithium group vs. 0.76 ± 0.30 Gy/MBq for the control group, *p* = 0.020). Thus, the use of lithium augmentation may allow the administered activity to be reduced without notably worsening the clinical outcome. This observation suggests a very important benefit of lithium augmentation in minimizing radiation exposure in patients with GD, especially that of young patients with long potential latency periods to develop radiation-induced toxicity, without sacrificing the anti-hyperthyroid efficacy of the RAI.

## Limitations

Certain limitations of the present analysis should be noted. First, neither the comparisons of lithium-augmented vs. standard RAI nor the two lithium regimens were prospective or randomized. Second, intergroup differences in the administered activity were attributable to a change in the formula for calculating individualized activities, i.e., the use of a longer, empirically derived value rather than published data on radioiodine effective half-life; activities were not formulated with potential effects of lithium in mind. Third, data on cure rates were not available for all patients (10% of the lithium group and 22% of the control group lost to follow-up). Fourth, the data on cure rates came from a relatively short follow-up of ~6 months. Hence, as noted earlier, this analysis should be regarded as exploratory. Our findings should be confirmed in a prospective, randomized controlled trial with careful evaluation of RAI efficacy after a longer follow-up.

## Conclusion

This retrospective study on patients with GD suggests that lithium administered in relatively low doses for relatively short periods could help to achieve higher RAIU in the thyroid, and higher absorbed dose per administered activity within that organ, from RAI. Thus, lithium may increase the effectiveness of RAI therapy. Alternatively, lithium augmentation could help reduce the administered radioiodine activity without lowering the efficacy of RAI. Our observations should be confirmed in a prospective randomized, controlled study.

## Data availability statement

The raw data supporting the conclusions of this article will be made available by the authors, without undue reservation.

## Ethics statement

No Ethics Committee approval was required for the present analysis, because it was an observational study using de-identified data. The studies were conducted in accordance with the local legislation and institutional requirements. The participants provided their written informed consent to participate in this study.

## Author contributions

FK: Conceptualization, Methodology, Supervision, Validation, Writing – original draft, Writing – review & editing. AS-S: Conceptualization, Formal analysis, Investigation, Software, Writing – original draft. LR: Conceptualization, Data curation, Formal analysis, Methodology, Writing – original draft. CB: Investigation, Software, Visualization, Writing – review & editing. FR: Investigation, Software, Supervision, Validation, Writing – review & editing. SE: Conceptualization, Methodology, Validation, Writing – review & editing.
